# Simulated Root Resorption: A New Study Model

**Published:** 2008-01-10

**Authors:** Jamileh Ghoddusi, Saeed Asgary, Masoud Parirokh, Mohammad Jafar Eghbal, Mahdi Vatanpour, Fatemeh Shahrami

**Affiliations:** 1 *Department of Endodontics, Dental Research Center, Faculty of Dentistry, Mashad University of Medical Sciences, Mashad, Iran*; 2 *Department of Endodontics, Iranian Center for Endodontic Research, Dental Research Center, Shahid Beheshti University of Medical Sciences, Tehran, Iran*; 3 *Department of Endodontics, Dental Research Center, Faculty of Dentistry, Kerman University of Medical Sciences, Kerman, Iran*; 4 *Department of Endodontics, Faculty of Dentistry, Azad University of Medical Sciences, Tehran, Iran*; 5 *Department of Endodontics, Faculty of Dentistry, Mashad University of Medical Sciences, Mashad, Iran*

**Keywords:** Dental Model, Root Resorption, Simulation

## Abstract

**INTRODUCTION:** The aim of this innovative study was to regenerate a condition that makes it possible to carry out researches in the field on a variety of resorption.

**MATERIALS AND METHODS:** In order to develop apical resorption, the root canals of selected teeth were instrumented and then drilled with #4 Gates Glidden drills. In the next stage, the teeth were submerged in melted rose wax up to 3 mm to the apex. The waxed teeth were submerged in 20% sulfuric acid for 4 days. After that, all samples were evaluated under stereomicroscope and also the SEM.

**RESULTS:** Images showed areas with different pattern of resorption in root apex and entire root canals in all samples.

**CONCLUSION:** Simulation of the root resorption can be helpful in many experimental studies.

## INTRODUCTION

It is obvious that the development of medical sciences is in debt to different clinical and experimental researches done in the field of science. With the expansion of the science field and its specialization of each individual field of science, these fields are helping each other to create research patterns. In general, there are two groups of studies commonly in use: clinical (*in vivo*) and laboratory studies (*ex vivo*). As clinical studies conform to real clinical conditions, they have higher validity (external validity) in the realm of application. On the other hand, because of the effects of several interfering variables, it is possible for various deficiencies to exist. Therefore, in order to eliminate these disturbing variables; and to control and evaluate desirable variables, in vitro studies have been exclusively designed. In conducting these in vitro studies, attempts must be done to prevent the affects of different side factors and to solely control those independent and dependent variables. Moreover, conducting number of experiments pertaining to equipment and material on laboratory animals and humans requires success in vitro tests. These types of tests have high internal validity, but conducting some of these tests may present difficult conditions because real conditions of test regeneration must be considered. Until now, various experimental models have been developed in the research realm of endodontic science.

Nattress *et al. *in 1997 (1), for the purpose of Endodontical technical training used extracted teeth that had been previously waxed. In 2007 Plotino *et al. *(2), used extracted teeth to check the function of rotary files. Romero et al in 2000 (3), for the purpose of recreating teeth conditions, with the help of silicone gel, designed and regenerated artificial PDL. With the purpose of testing cyclic fatigue of rotating files, others used stainless steel tubes (4). Ai *et al. *in 2003 (5), with the help of alginate and in 2007, Topuz *et al. *(6), with saline solution created a condition that was capable of transmitting electricity similar to mouth tissues. In order to perform a precision test they used apex locator equipment. Several researchers also used computer patterns made by different software such as ANSIS and with the help of finite elements evaluated the tension and spread of forces in teeth (7-8). Resembling previous studies, many studies used extracted teeth. However, for conducting studies on some conditions, such as internal and external resorption, it is impossible to use extracted teeth because obtaining teeth with those conditions are rare.

**Figure 1:**
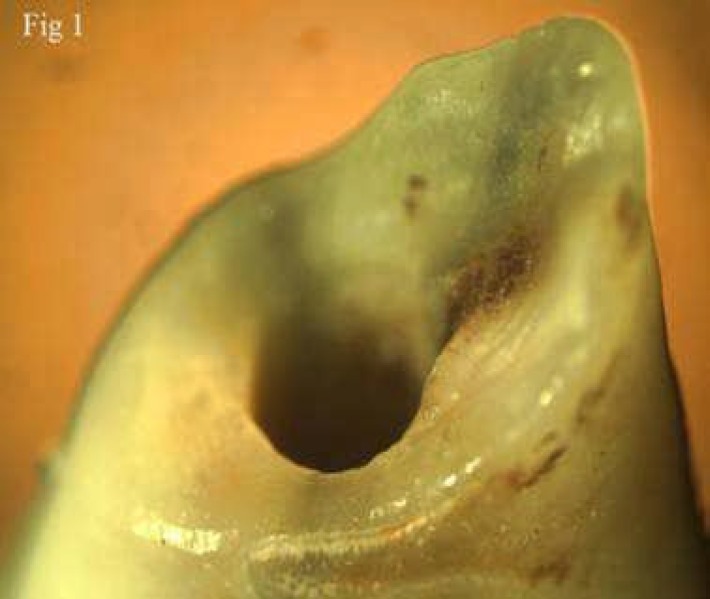
Stereomicroscopic feature of simulated root resorption

The goal of this innovative study was to regenerate a condition that makes it possible to carry out a variety of researches in the field on a variety of simulated resorption conditions.

## MATERIALS AND METHODS

The protocol of this study was approved by ethical committee of Australian National University (Protocol 2006/213). In order to conduct this research project, first twenty extracted human single root canal teeth were gathered that were pulled out for various reasons, such as periodontal disorders. Then in order to develop apical resorption, which had approximately similar sizes, we drilled the ends with a #4 Gates Glidden drill (Maillefer, Dentsply, Switzerland). Of course, the size was goal. In the next stage, it was necessary to develop those disorders similar to what occurred in the resorption site. Realizing the large reserve of calcium in teeth, 20% sulfuric acid was the best material to aid in this purpose. However, to limit the development process of resorption in the apical end, the teeth were submerged in melted rose wax up to 3 mm to the apex. The waxed teeth were submerged in 20% sulfuric acid for 4 days. Following this period all samples were evaluated under stereomicroscope (Nikon SMZ10, Tokyo, Japan) to check the apical resorption. The teeth were then coated with thin layer of gold and examined with low magnification of scanning electron microscope (Hitachi S2250-N, conventional and low vacuum SEM, Japan) operating at 20 KV.

**Figure 2:**
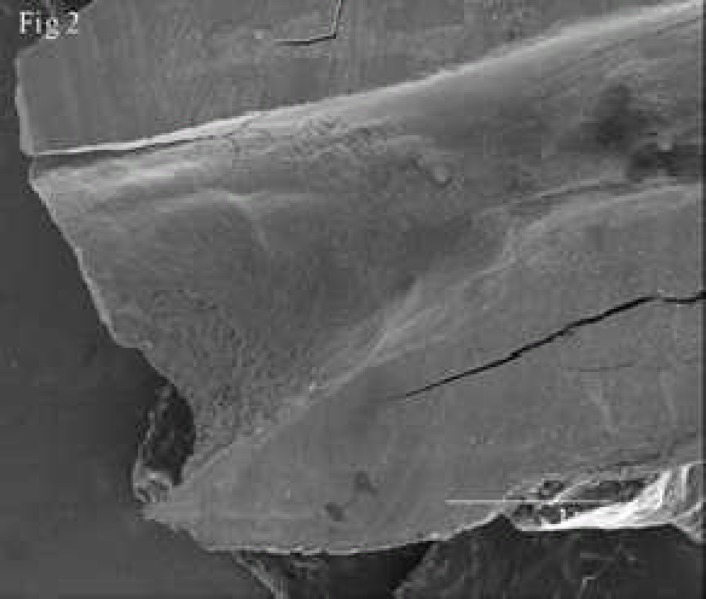
A low magnification SEM shows the resorption in root end and entire canal in one of the samples

## RESULTS

**Figure 3:**
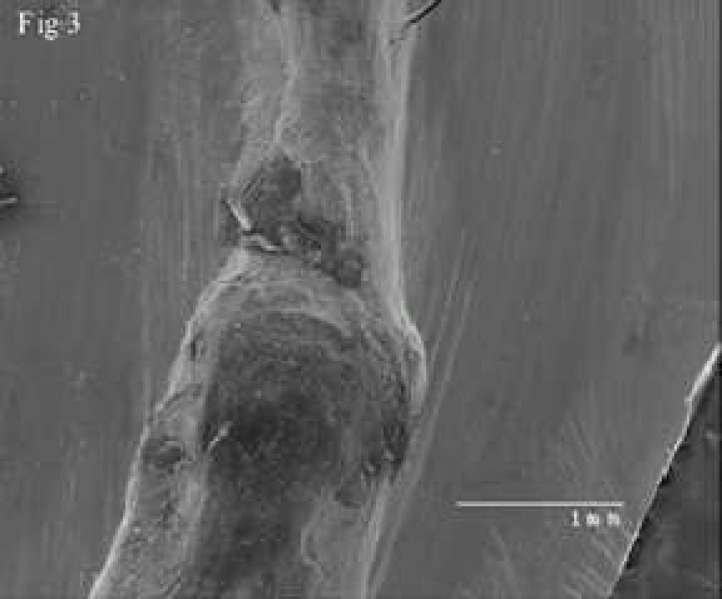
A low magnification scanning electron micrograph shows the resorption in entire root canal in one sample

Resorptions were observed in apical region and also in entire root canals of all prepared samples. Simulated resorbed walls were expectedly irregular and occurred in different patterns (Figure 1), (Figure 2), (Figure 3).

## DISCUSSION

Since this is a unique and innovative method, similar studies do not exist; hence we are not able to compare the results of our study. Nevertheless, regarding the various stages that have been conducted some points should be considered. As stated previously, to prepare the apical region, a #4 Gates Glidden drill was used. The ability to recreate similarity to the dimensions of a common resorption was the reason for choosing this drill size. Also, if the preparation of teeth with resorption were an initiation for conducting other studies it was essential to have approximate size and dimension similarity between samples in order for the size factor of the resorption region not to be an interfering variable. Therefore, it would be possible to prepare size or even yet apical surface forms based on other primary study goals. For example, if necessary it would be possible to produce a bevel and cut the apical root end resembling a procedure in Endodontic surgery in order to create resorption changes.

In this study, in order to control the resorption region and contain it in favorable apical areas all teeth walls were covered with modeling wax. Based on initial tests we discovered that sulfuric acid did not have any resolving effects on waxes. The use of melted wax and the flow of wax throughout teeth surfaces caused a suitable seal and consequently strong teeth protection against acid. We were not able to do this using nail polish and epoxy resin, because these products did not have enough resistance to the acid and their integration was lost and therefore the sulfuric acid inadvertently penetrated teeth walls.

The important point of this innovative process was the type of acid that was chosen to be used. In previously published studies (9-10) many of these researchers used a variety of acids such as chloric acid, nitric acid and formic acid, in different consistencies for decalcification of teeth in order to make the teeth transparent. Unfortunately, these acids used in those consistencies, were not able to eliminate organic tissues such as collagen and they could only eliminate mineral matter on teeth and the structure of collagen remained; and although they became soft and flexible, they retained their primary shape. However, in the process of this study it was necessary to take samples of matrix and mineral materials, which also included organic and mineral tissues. While conducting various tests it became obvious that sulfuric acid was more able to carry out this targeted action. It worths mentioning that increasing the consistency of acids used in the process of teeth clearing caused complete decomposition of teeth and prevented the taking of matrix samples.

Regarding the consistency used, it must be noted that this consistency had a direct relation with the duration of developing resorption.

While experimenting with different consistencies, 10%, 20%, 30%, it appeared that 20% was the best consistency, when used in a suitable time frame, for controlling the total depth of developed resorption. The reason is that increasing the consistency expedited the development of resorption and therefore the researcher would have lost control of developing the desired patterns. In this study due to the effect of irregular acid resorption on external and internal walls, the apical region was prepared; therefore, regular resorption patterns similar to real cases occurred.

At the end, this is noteworthy that these methods can be used for other studies and are not exclusive for apical region. Moreover, the mentioned procedure can be also be used to resemble a variety of resorptions such as surface cervical resorption, external and internal resorption, and resorptions causing perforations in diverse areas.

## CONCLUSION

According to our finding we conclude that the present method for simulation of the root resorption can be used in many experimental studies.
